# Masked mRNA is stored with aggregated nuclear speckles and its asymmetric redistribution requires a homolog of mago nashi

**DOI:** 10.1186/1471-2121-12-45

**Published:** 2011-10-13

**Authors:** Thomas C Boothby, Stephen M Wolniak

**Affiliations:** 1 Department of Cell Biology and Molecular Genetics, University of Maryland, College Park, MD 20742, USA

## Abstract

**Background:**

Many rapidly developing systems rely on the regulated translation of stored transcripts for the formation of new proteins essential for morphogenesis. The microspores of the water fern *Marsilea vestita *dehydrate as they mature. During this process both mRNA and proteins required for subsequent development are stored within the microspores as they become fully desiccated and enter into senescence. At this point microspores become transcriptionally silent and remain so upon rehydration and for the remainder of spermatogenesis. Transcriptional silencing coupled with the translation of preformed RNA makes the microspore of *M. vestita *a useful system in which to study post-transcriptional regulation of RNA.

**Results:**

We have characterized the distribution of mRNA as well as several conserved markers of subnuclear bodies within the nuclei of desiccating spores. During this period, nuclear speckles containing RNA were seen to aggregate forming a single large coalescence. We found that aggregated speckles contain several masked mRNA species known to be essential for spermatogenesis. During spermatogenesis masked mRNA and associated speckle proteins were shown to fragment and asymmetrically localize to spermatogenous but not sterile cells. This asymmetric localization was disrupted by RNAi knockdown of the *Marsilea *homolog of the Exon Junction Complex core component Mago nashi.

**Conclusions:**

A subset of masked mRNA is stored in association with nuclear speckles during the dormant phase of microspore development in *M. vestita*. The asymmetric distribution of specific mRNAs to spermatogenous but not sterile cells mirrors their translational activities and appears to require the EJC or EJC components. This suggests a novel role for nuclear speckles in the post-transcriptional regulation of transcripts.

## Background

*M. vestita *is an aquatic, heterosporous water fern whose sporophyte resembles a four-leaf clover. Its microspores and megaspores are meiotic products that desiccate and become dormant after they are formed. Upon rehydration, the microspores develop rapidly to produce male gametophytes that make multiciliated spermatozoids [for review, [1].

Like other rapidly developing systems [2-7], male gametophyte development and spermatid differentiation of *M. vestita *depends on little or no new transcription. The microspore becomes transcriptionally silent during its desiccation and remains so upon rehydration and initiation of spermatogenesis [8]. Therefore, transcriptional activity essential for gametophyte development occurs prior to spore desiccation, and after spore hydration, spermiogenesis relies on the translation of stored mRNAs [for review, [1]. In this system, the mobilization, distribution and processing of stored mRNAs in the gametophyte underlies patterns of rapid development.

Not surprisingly, the translation of specific stored transcripts is under tight temporal and spatial control [9-12]. One example of this spatial and temporal regulation of stored transcripts is centrin mRNA. Centrin is a calcium-binding phosphoprotein that has been shown to be essential in motile apparatus formation in the microspore of *M. vestita *[9]. Centrin mRNA is uniformly distributed throughout the cytoplasm of the microspore from the onset of gametophyte development, but centrin protein levels are barely detectable during of the first four hours after the spores are hydrated. Beyond that time point, centrin protein levels increase dramatically, but only in the spermatogenous cells, where they remain elevated through the completion of gamete formation [13,9,10]. Thus, the translational capacity for centrin protein synthesis is asymmetric, because centrin mRNA is present in the cytoplasm of both sterile and spermatogenous cells in the gametophyte, but centrin is translated only in spermatogenous cells [12]. Centrin RNA was examined in this study because of the extensive amount of preexisting knowledge regarding its spatial and temporal dynamics during microspore development [for review see: [1]. Similarly, temporal and spatial control over translation has been observed for a number of other transcripts [12] and proteins [10] in these gametophytes.

An important mechanism regulating gametophyte development is the unmasking of stored transcripts for translation [for review, [14]. Within this context we define "masked RNA" as mRNA whose translational state is initially inhibited, but later is "unmasked" to become translationally competent. This pool of masked mRNA is stored in the nucleus of the desiccated spore [15]. We refer to mRNA that is uniformly distributed in the cytoplasm of all cell types in the gametophyte, but does not appear to be translated at anytime during development as quiescent cytoplasmic mRNA (qc-mRNA).

Recently, we found that the polyamine, spermidine (SPD), acts as a temporal regulator for releasing the masked, stored transcripts in the gametophyte [1,15]. Exogenous additions of SPD and other polyamines at the time of spore hydration cause the precocious unmasking of spermidine synthase (SPDS) mRNA in addition to other masked transcripts including centrin, PRP-19, and gamma-tubulin [15]. High concentrations of SPD also arrest division cycles, presumably because of premature transcript unmasking. Precociously unmasked transcripts display an intriguing pattern of distribution, and appear as distinct particles in the nucleus [15]. These findings led us to hypothesize that a subset of masked transcripts is stored within the nucleus of the microspore and that the temporal regulation of these transcripts is dependent on unmasking as a prerequisite for translation essential to the proper completion of spermatogenesis. Since masked transcripts appear to be stored within the nucleoplasm of the microspore as it undergoes desiccation, we were also interested if these masked transcripts are associated with known nuclear bodies.

Nuclear speckles are small aggregations (~1 μm) of 20-25 nm granules that occupy the interchromatin space of many eukaryotic nuclei [16]. Several types of pre-mRNA processing proteins are constituents of nuclear speckles [17], and speckles also contain a subset of poly(A)+ RNA [18-20]. Within the interchromatin space of the nucleus, speckles are often localized adjacent to genes with high transcriptional activity [21-26]. While a direct role for nuclear speckles in transcription and post-transcriptional modification has neither been confirmed nor disproven definitively, several lines of evidence suggest a role for speckles in the transport and splicing of pre-mRNA [for review, [27]. Interestingly, upon an inhibition of transcription, speckles have been observed to enlarge and assume a rounded morphology, which has been suggested to result from the storage of pre-mRNA splicing factors [28-30]. In addition to pre-mRNA splicing factors, a subset of poly(A)+ remains associated with enlarged nuclear speckles within the nucleus under conditions where transcription has been inhibited [18]. The purpose of this nuclear retention of RNA is unknown.

Stress conditions such as hypoxia, inhibition of respiration, transcription, phosphorylation and ethanol treatment have been shown to cause the sequestration of the Exon Junction Complex (EJC) core component eIF4A-III to several subnuclear structures including nuclear speckles [31,32]. It is likely that mRNA associated with these sequestered components will be retained within the nucleus and not translated. It is likely that the association of core components of the EJC with subnuclear RNA and/or nuclear speckles could play a vital role in regulating a subset of cellular processes. Previously experiments have demonstrated the ubiquitous expression of EJC core component Mago nashi in other plant systems and that the loss of this expression has extensive effects on development [33]. Since the microspore of *M. vestita *is a transcriptionally silent system that relies on the translation of stored mRNA after the spore is released from desiccation, and contains a subset of nuclear localized masked transcripts, we suspected that examining nuclear speckle dynamics might lead to insights into developmental control in the maturing gametophyte.

In this study, we have examined nuclear speckle dynamics during microspore entry into dormancy and transcriptional quiescence. We show that in addition to cytoplasmic stores, aggregated nuclear speckles serve as sites of poly(A)+ RNA storage. We developed a novel variation on fluorescence *in situ *hybridization (FISH), in an assay designed to distinguish between masked and unmasked (qc-mRNA) populations of the same transcripts in fixed cells. We demonstrate the utility of this assay by tracking the movements of specific transcripts initially stored in association with nuclear speckles into the cytoplasm of the antheridial mother cell. We show subsequent movements of masked transcripts into spermatogenous, but not jacket cells of the developing gametophyte, and that this asymmetry may be regulated via the EJC component Mv-Mago. The asymmetric distribution of one of these transcripts (centrin) mirrors its pattern of translation and presents a likely mechanism for post-transcriptional regulation essential for cell fate determination during the rapid and precise process of spermatogenesis in *M. vestita*.

## Results

### Nuclear speckles coalesce during desiccation and transcriptional silencing to form a single nuclear speckle aggregate

We examined nuclear speckle dynamics during microspore desiccation, since this is the developmental period when the gametophyte becomes transcriptionally silent. We harvested microspores from green, mostly-submerged sporophytes prior to the beginning of dehydration and subsequently every 2 weeks thereafter, as the ponds containing the sporophytes were allowed to dry out.

For all experiments using sectioned material presented here, thousands of microspores were grown, fixed, and embedded. Each experiment was conducted independently a minimum of 2 times. Between 30-40 sections were used per slide so that many hundreds of sectioned spores were observed for each experimental trial, of which a no less then 20 representative photographs were taken to ensure accurate analysis. The number of independent trails per experiment, and total number of photographs taken for each set of experiments are listed in Additional file 1.

**Figure 1:**
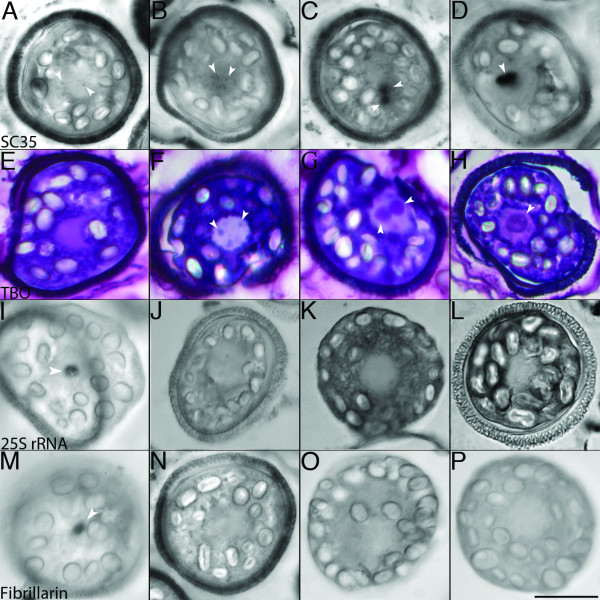
**Nuclear speckles aggregate during desiccation of the microspore of *M. vestita***. Bright field microscopy: **a-d**, Labeling of microspores with SC-35 mAb against nuclear speckle marker SC35. **e-h**, TBO staining. **i-l**, ISH with probe for 25S rRNA. **m-p**, Labeling of microspores with mAb 72B9 against nucleolar marker Fibrillarin. Microspores were harvested from plants that were watered (**a**, **e**, **i**, &**m**), had not been watered for 2 weeks (**b**, **f**, **j**, &**n)**, had not been watered for 4 weeks (**c**, **g**, **k**, &**o) **and had not been watered for 6 weeks (**d**, **h**, **l**, &**p)**. Arrows denote subnuclear aggregations. Bar = 25 μm.

SC35 is a non-snRNP splicing factor that is often used as a specific marker of nuclear speckles [for review, [34]. We used the distribution of SC35 protein to assay for the presence of nuclear speckles and to assess putative speckle dynamics in drying spores (Figure 1a-1d). Because there is a high level of autofluorescence in drying and desiccated microspores, making immunofluorescence impossible, we instead used alkaline phosphatase conjugated secondary antibodies and the nitro-blue tetrazolium and 5-bromo-4-chloro-3-indolyl phosphate detection method to label cells during this stage of development. Prior to desiccation, SC35 exhibited a typical speckled pattern throughout the nucleus (Figure 1a). After 2 weeks of sporophyte drying, these speckles had increased in labeling intensity (Figure 1b). By 4 weeks of drying, the speckled appearance of SC35 labeling appeared to have been replaced by larger aggregates of the protein within the nucleus (Figure 1c). With total desiccation of the spore, SC35 was detectable as a single, large subnuclear aggregation (Figure 1d).

We used the metachromatic dye Toluidine Blue O (TBO; Figure 1e-1h) to confirm our SC35 nuclear speckle labeling. In spores harvested prior to desiccation, TBO staining did not reveal the presence of any accumulated subnuclear material in the microspores (Figure 1e). In samples taken from ponds that had not received water for 2 weeks, TBO staining revealed small, multiple subnuclear aggregations in microspore nuclei (Figure 1f). As the desiccation process continued, these small aggregations coalesced into larger particles (Figure 1g, h), until a single large aggregation occupied most of the volume of the nucleoplasm.

Since the single aggregate present in dry spores superficially resembles a nucleolus, we used markers known to associate with nucleoli to confirm we were looking at aggregated speckles. To distinguish between speckles and nucleoli, we analyzed 25S rRNA distributions by in situ hybridization (ISH) in the desiccating microspore, which we expected to label large nucleoli. This rRNA was present as a conspicuous nuclear particle (Figure 1i), and exhibited some cytoplasmic staining in the microspores prior to the onset of desiccation. Nuclear 25S rRNA persisted during the first 2 weeks of desiccation (Figure 1j) but became undetectable thereafter and for the remainder of the dehydration process (Figure 1k, l). Concurrent with the loss of nuclear 25S rRNA, levels of 25S rRNA increased in the cytoplasm as dehydration progressed (Figure 1i-1l), consistent with the export of newlyassembled ribosomes to the cytoplasm. Similarly, Fibrillarin, a rRNA processing factor and nucleolar marker, was apparent in a conspicuous nuclear inclusion (Figure 1m) identical in morphology to 25S rRNA ISH patterns obtained at this time point (compare with 1i). The abundance of Fibrillarin within desiccating microspores declined over time, and was weakly detectable up to but not after 2 weeks of drying (Figure 1n-1p). The disappearance of the nucleolar markers from the nuclei of desiccating microspores shows that ribosomal synthesis reaches completion before the spores are totally dry. As the nucleoli become less prominent, the nuclear speckles become more conspicuous, thereby revealing a developmental shift from rRNA synthesis to pre-mRNA synthesis. Moreover, continued desiccation and a transition to transcriptional quiescence triggers the aggregation of nuclear speckles in the microspore of *M. vestita*.

### Nuclear speckles remain aggregated in the newly hydrated microspore

**Figure 2:**
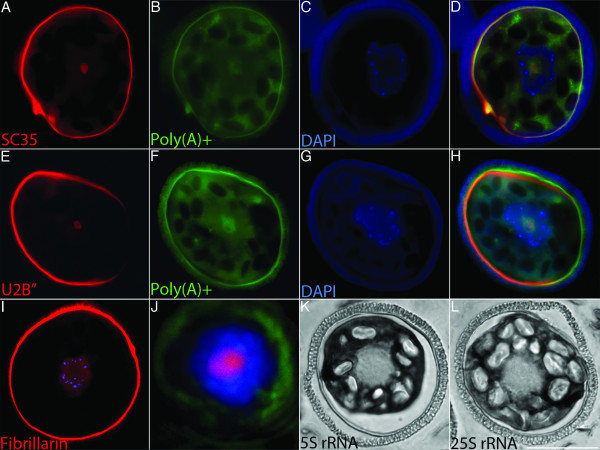
**Nuclear speckles remain aggregated within the nuclei of newly hydrated microspores**. **a**-**i**, **k**, and **l**, microspores were fixed and sectioned after 30 minutes of development. **a**-**d**, dual detection of (**a**) SC35 (red) and (**b**) Poly(A)+ RNA (green). **c**, DAPI (blue). **d**, Merge of **a**-**c**. **e**-**h**, Dual detection of (**e**) U2B" (red) and (**f**) Poly(A)+ RNA (green). **g**, DAPI (blue). **h**, Merge of **e**-**g**. **i**, Microspore labeled with monoclonal antibody 72B9 against Fibrillarin (red) and counter stained with DAPI (blue). **j**, Monoclonal antibody 72B9 labeling (red) of the nucleolus in a sporophytic cell, counter stained with DAPI (blue) and overlain on the phase contrast image (green) of the gametophyte. **k**, Representative 5S rRNA ISH labeling of the cytoplasm of a microspore (dark staining). **l**, Representative 25S rRNA ISH labeling of the cytoplasm of a microspore (dark staining). Bar = 25 μm.

Rehydration of the microspore triggers the commencement of spermatogenesis, but does not disrupt transcriptional silencing. We were interested in seeing if speckles remained aggregated after microspore rehydration. We examined the distribution of nuclear speckle markers, SC35 and U2B", a snRNP splicing factor known to be present in nuclear speckles. Since a subset of poly(A)+ RNA is retained in speckles during transcriptional inhibition [18], we used a biotinylated poly(T) probe for Poly(A)+ RNA as an additional marker for aggregated speckles. SC35 protein and poly(A)+ RNA localized as an aggregate within the nucleus of newly hydrated microspores (Figure 2a-2d). U2B" protein also overlapped with poly(A)+ RNA within the nucleus (Figure 2e-2h).

Similar to our observations of later stages of spore desiccation, nucleolar markers did not reveal the presence of a nucleolus in the rehydrated microspores. As expected, Fibrillarin was barely detectable within the nucleus of newly hydrated microspores and appeared diffuse throughout the nucleoplasm (Figure 2i), whereas anti-Fibrillarin antibody labeled the nucleoli of sporophytic cells (Figure 2j) and microspores prior to desiccation (Figure 1m) robustly label nucleoli, demonstrating the efficacy of this antibody in *M. vestita*. In ISH assays, 5S and 25S rRNA were not detected within the nuclei but were abundant throughout the cytoplasm of newly hydrated microspores (Figure 2k, l). These data confirm that nuclear speckles remain aggregated within the nucleus of newly hydrated microspores of *M. vestita*, and, as seen in mammalian systems [18], these enlarged speckles contain a subset of poly(A)+ RNA.

### Nuclear poly(A)+ RNA leaves the nucleus during the first division

**Figure 3:**
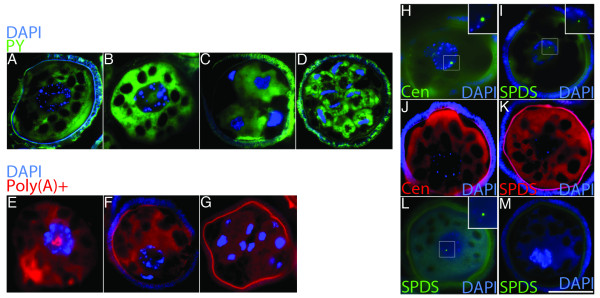
**Masked mRNA is stored within the microspore of *M. vestita***. **a**, Microspore fixed after 30 minutes of development, double-stained with DAPI (blue) and PY (green). **b**, Microspore fixed after 45 minutes of development (just prior to the prothallial division) double-stained with DAPI (blue) PY (green). **c**, Microspore fixed after 1.5 hours of development double- stained with DAPI (blue) PY (green). **d**, Microspore fixed after 5 hours of development double-stained with DAPI (blue) PY (green). **e**-**g**, Distribution of Poly(A)+ RNA during spermatogenesis. A biotinylated poly(T) probe (red) was used for FISH on microspores fixed at (**e**) 30 minutes, (**f**) 1 hour, and (**g**) 4 hours of development. FISH probes were detected using avidin bound TexasRed. **h-m**, Microspores fixed after 30 minutes of development. **h**, FISH using 25mer biotinylated centrin specific probes, and detected using avidin bound Fluorescein (green). **i**, FISH using 25mer biotinylated SPDS specific probes, and detected using avidin bound Fluorescein (green). **j**, FISH using traditional biotinylated centrin specific probes, and detected using avidin bound TexasRed (red). **k**, FISH using traditional biotinylated SPDS specific probes, and detected using avidin bound TexasRed (red). **l**-**m**, Microspores pretreated with (**l**) DNase or (**m**) RNase and incubated with 25mer biotinylated SPDS probes, which were detected using avidin bound Fluorescein (green). Bar = 25 μm.

Earlier work showed that spermatogenesis in *M. vestita *relies on the translation of stored mRNA [8-10,12,13], but this translation cannot take place within the first 30 minutes after spore hydration [8]. We were interested in whether poly(A)+ RNA present in the nuclear speckle aggregate could be utilized by the microspore after this initial period of quiescence in early gametophyte development. A prerequisite for the translation of speckle associated RNA would be its movement into the cytosol. We studied the localization and storage of masked mRNA in microspores after developing a fluorescent DNA/RNA differential detection assay based on the methyl green/pyronin Y histochemical staining technique. Histochemical dyes (such as pyronin Y) lack exclusive specificity for RNA, so off-target binding to other nucleic acids can cause erroneous results [35]. One solution to this problem is to use a DNA-binding dye, such as methyl green, as a competitor dye in conjunction with an RNA-binding dye, such as pyronin Y, with the aim of eliminating or decreasing off target binding [36]. We found that the commonly used DNA preferential method employing methyl green/pyronin Y staining is ineffective in *M. vestita *because the chromatin becomes tightly condensed in the spermatids. We recently showed that DAPI labels chromatin in the developing spermatids of *M. vestita *[37], so we developed an assay similar to methyl green/pyronin Y staining by substituting DAPI for methyl green. This substitution greatly reduced the off-target labeling of both DNA and RNA (Additional file 2) allowing us to examine RNA distributions during microspore desiccation (Additional file 3, 4, 5) and gametophyte development (Figure 3).

We used this dual staining assay to assess the distribution of stored RNA during male gamete development (Figure 3a-3d). At all time points examined (30 minutes, 45 minutes, 1.5 hours, and 5 hours), high concentrations of RNA were seen within the cytoplasm of both spermatogenous and sterile cells. Prior to the first division, a large aggregation of RNA was observed within the nuclei of newly hydrated microspores (Figure 3a). After the first division cycle had occurred (Figure 3c, d), the large RNA aggregate was no longer detected within the nucleus, and PY staining was absent from the nucleoplasm (Figure 3c, d, Additional file 6).

To confirm these results we used our biotinylated poly(T) probe to detect poly(A)+ RNA movements during development. At 30 minutes of development, a large aggregate of poly(A)+ RNA was seen within the nucleoplasm of undivided microspores (Figure 3e). At the completion of the first (prothallial) division, poly(A)+ RNA was no longer detected within the nuclei of cells (Figure 3f) and this lack of nucleoplasmic poly(A)+ RNA persisted through all the division cycles (Figure 3g). Poly(A)+ RNA relocalized in an identical pattern as total RNA (Figure 3e-3g compare with 3a-3d); poly(A)+ RNA that had been stored in association with aggregated nuclear speckles exits the nucleus during the first division of the gametophyte. This is the earliest time point in development when proteins can be translated *in vitro *from gametophyte mRNA isolates [8].

### Masked mRNA species localize to discrete foci within the nuclear speckle aggregate

Since precociously unmasked RNAs are detectable within the nucleus of the newly hydrated spore after 10 mM polyamine additions [15], we reasoned that masked transcripts could constitute a subset of speckle associated poly(A)+ RNA. Since additions of SPD cause mitotic arrest and the precocious unmasking of transcripts [15], it is reasonable to assume that if these transcripts are associated with nuclear speckles, then SPD should perturb speckle aggregation within the microspore nucleus. We tested this hypothesis by adding 10 mM SPD to microspores, which were then allowed to develop for 4 hours. The effect of SPD on speckle aggregation was examined during PY-DAPI labeling, and we found that SPD causes the partial or complete dissociation of speckles (Additional file 7, 8). In cases of partial dissociation (Additional file 7) the subnuclear PY signal was observed as one or more amorphous masses within the nucleoplasm of the microspore. Total dissociation of the speckle aggregate was seen as the loss of subnuclear organization; the speckle aggregate no longer occupied a defined central portion of the nucleoplasm but rather, the PY signal was dispersed throughout the nucleus but apparently contained by the nuclear envelope (Additional file 8).

To confirm that masked transcripts are associated with aggregated speckles, and then, to examine their role in spermatogenesis, it was essential to find a method for visualizing specific masked transcripts in unperturbed microspores. We found that traditional ISH and FISH methods work well for determining the localization of qc-mRNA transcripts, but these strategies fail to label masked transcripts [12,15] present in the speckles, presumably because masking agents obscure hybridization sites. We found that a 'short' 30 base poly(T) probe would label subnuclear RNA in the newly hydrated spores (Figure 2b, e3e), thereby suggesting that small FISH probes might reveal the localization patterns of both masked and qc-mRNA by hybridizing between masking agents.

We made short 25mer biotinylated DNA probes complementary to SPDS and centrin mRNAs, transcripts that become detectable by ISH within the nucleus after treatments of spores with 10 mM SPD [15]. The 25mer probes were added to sections of fixed gametophytes that had been developing for 30 minutes (Figures 3h, i). These 25mer probes showed diffuse cytoplasmic labeling in addition to intense subnuclear labeling at conspicuous foci in newly hydrated microspores (Figures 3h, i). Longer hybridization probes derived from SPDS and centrin were incubated with successive sections from the same specimen block, containing the same gametophytes (Figure 3j, k). The longer probes hybridized with qc-mRNAs present throughout the cytoplasm, but these transcripts were undetectable in the nuclei of the cells. Samples treated with DNase and assayed with "short" probes retained their cytoplasmic and subnuclear hybridization patterns for the tested transcripts (Figure 3l), while samples pretreated with RNase before short probe hybridization lacked any detectable RNA labeling (Figure 3m). The fluorescent foci detectable with short-probe hybridizations were usually centrally situated in the nucleus and did not overlap with any of the chromosomes (Additional file 9). Thus, the short FISH probes are not hybridizing to chromosomes.

**Figure 4:**
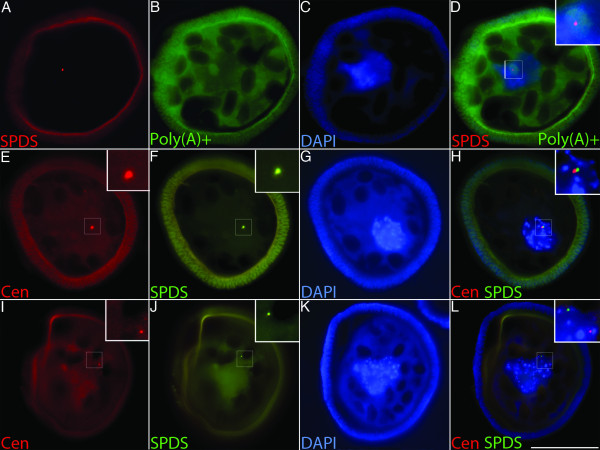
**Subnuclear poly(A)+ RNA localizes with discrete foci of masked mRNA in the microspore of *M. vestita***. **a**-**l**, Developing microspores were fixed at 30 minutes of development. **a**-**d**, Dual FISH labeling of microspores with (**a**) 25mer biotinylated probe against SPDS (red) and (**b**) a poly(T) probe (green). SPDS probe was detected using avidin conjugated TexasRed (red) (**a**). An avidin/biotin blocking step was performed, followed by labeling and detection of the Poly(T) probe using an avidin conjugated Fluorescein (green) (**a**). **c**, DAPI (blue). **d**, Merge of **a**-**c**; inset shows enlarged view of subnuclear poly(A)+ RNA (green) and masked SPDS (red). 25mer biotinylated probes for centrin (red) (**e **&**i**) and SPDS (green) (**f **&**j**) were used to label sections sequentially. Centrin probe was detected using avidin conjugated TexasRed (red) (**e **&**i**). An avidin/biotin blocking step was performed, followed by labeling and detection of SPDS probe using an avidin conjugated Fluorescein (green) (**f **&**j**). Sections were counter stained with DAPI (blue) (**g **&**k**). Centrin (red), SPDS (green), and DAPI (blue) labeling were merged in **h **and **l**, insets show foci of short probe hybridization. Bar = 25 μm.

Dual FISH labeling with a poly(T) probe and a 25mer SPDS probe showed that foci of specific masked transcripts localize with nuclear speckle aggregate-associated poly(A)+ RNA (Figure 4a-4d). Double labeling of samples with short probes for SPDS and centrin showed distinct foci for each transcript within the nucleoplasm (Figure 4e-4l). These data taken with our previous finding (that polyamine additions can cause the unmasking of subnuclear RNA [15]) demonstrate that masked mRNAs are stored in discrete foci associated with aggregated nuclear speckles, and that the addition of SPD disrupts both the aggregation of nuclear speckles and the masking of these mRNAs.

### RNA and protein components of the nuclear speckle aggregate are asymmetrically redistributed to the cytoplasm of spermatogenous cells

**Figure 5:**
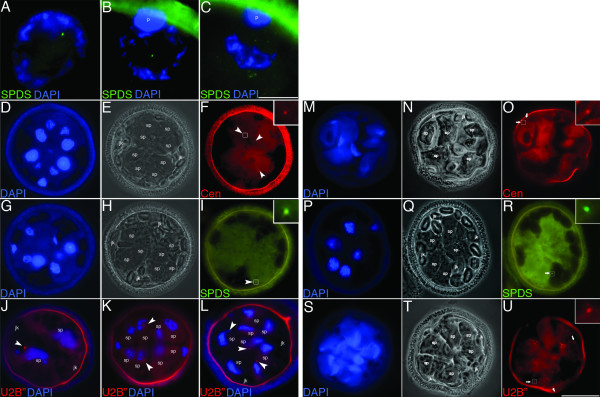
**Masked mRNAs and speckle markers enter the cytoplasm during the first division and Mago is required for their asymmetrically redistributed**. **a**-**c**, 25mer biotinylated probes directed against SPDS transcripts were hybridized and detected using avidin conjugated Fluorescein (green) and counter stained with DAPI (blue). Bar = 10 μm. **a**, Microspore prior to the prothallial division. **b**, Microspore during the prothallial division. **c**, Microspore just after completion of the prothallial division. The prothallial nucleus is denoted by "p" in **b **and **c**. **d-u**, "sp" denotes spermatogenous cells, "jk" denotes jacket cells, arrowheads mark foci of labeling within spermatogenous cells and arrows mark foci of labeling within jacket cells. **d-f**, Microspore fixed at 4 hours of development and probed with short FISH probes for masked centrin mRNA (red). **d**, DAPI (blue), **e **phase contrast, and **f **centrin FISH probes detected with TexasRed conjugated antibody (red). **g-i**, microspore fixed at 4 hours of development and probed with 25mer FISH probes for masked SPDS mRNA (green). **g**, DAPI (blue), **h **phase contrast, and **i **SPDS FISH probes detected with Fluorescein conjugated antibody (green). **j-k**, 4G3 labeling of U2B" protein (red) at (**j**) 2 hours, (**k**) 4 hours, and (**l**) 5 hours of development. Arrowheads denote cytoplasmic masked mRNA (**f **and **i**) and U2B" (**j**-**l**). **m-o**, Microspore subjected to Mv-Mago RNAi and fixed after 4 hours of development. (**m**) DAPI (blue), (**n**) phase contrast, (**o**) masked centrin transcripts (red) detected with 25mer biotinylated FISH probes. Arrows denote masked transcripts within jacket cells. Boxed regions enlarged in insets. Spermatogenous (sp) and jacket cells (jk) labeled in **n, q and t. p-r**, Microspore subjected to Mv-Mago RNAi and fixed after 4 hours of development. (**p**) DAPI (blue), (**q**) phase contrast, (**r**) masked SPDS transcripts (green) detected with 25mer biotinylated FISH probes. Arrows denote masked transcripts within jacket cells. Boxed regions enlarged in insets. **s**-**u**, Microspores subjected to Mv-Mago RNAi and fixed after 5 hours of development. (**s**) DAPI (blue), (**t**) phase contrast, (**u**) 4G3 labeling of U2B" (red). Arrow denotes U2B" within jacket cell. Bar = 25 μm.

We were interested in what happens to nuclear speckle components as they exit the nucleus, so we tracked the abundance and distribution of specific transcripts at various time points during spermatogenesis, starting as the gametophytes progressed through their first division cycle (Figure 5a-5c). Using short FISH probes specific for masked SPDS mRNA, we found that prior to the first division, masked SPDS transcripts were centrally localized within the nucleus (Figure 5a). As the first (prothallial) division approached, the nucleus became repositioned near the periphery of the microspore with the chromosomes to be apportioned to the prothallial cell arranged in a spherical array (Figure 5b). At this stage, masked SPDS transcripts were still encircled by the chromosomes that would later be segregated to the antheridial mother cell. As the prothallial division proceeded, masked SPDS transcripts relocated to a site in the cytosol adjacent to the antheridial mother cell nucleus (Figure 5c). Sections from the same sample blocks were made and assayed by FISH for SPDS qc-mRNA transcripts by using longer probes (Additional file 10). At all time points before, during, and after the prothallial division, cytoplasmic SPDS transcripts were abundant throughout the cytoplasm but not detectable in the nuclei (Additional file 10). Consistent with DAPI/PY staining and poly(A)+ FISH assays, it is evident that the subnuclear stores of masked mRNA exit the nucleus at the time of nuclear envelope breakdown during the first division, and are specifically passed on to the antheridial mother cell.

By the end of all nine mitotic division cycles in the gametophyte, small foci of both SPDS and centrin masked transcripts were apparent in the cytosol, adjacent to the nucleus of each spermatid (Figure 5d-5i). These fluorescent particles were absent in the adjacent jacket cells of the gametophyte.

Similar to masked mRNA, U2B" protein exhibited localized immunolabeling in aggregates within the antheridial initials (Figure 5j). By 4 hours of development, immunolabeling revealed that U2B" protein was dispersed as clusters of punctate foci adjacent to the nuclei of the spermatogenous cells (Figure 5k). During nuclear elongation in the maturing spermatids, the anti-U2B" antibody label predominantly colocalized with the ends of the elongating gamete nuclei (Figure 5l). Both masked mRNA and protein were initially localized within the nucleus of the microspore, and they assumed precise distributions only in maturing spermatogenous cells. Thus, nuclear speckle components are distributed asymmetrically to the cytoplasm of spermatogenous, but not, sterile cells. This asymmetry underlies cell fate determination in the gametophyte where a single cell in the microspore gives rise to two distinct cell types, sterile cells and spermatogenous cells, in a precise a temporal and spatial framework.

### Mv-Mago is required for the asymmetric distribution of components of the nuclear speckle aggregate

Previously, the RNAi-induced silencing of Mv-Mago, a homolog of the EJC component Mago nashi, was shown to deplete Mv-Mago levels within the spore as well as disrupt the endogenous asymmetry between spermatogenous and sterile cells in the developing male gametophyte of *M. vestita *[37]. A primary effect of depleting Mv-Mago mRNA was the loss of asymmetric centrin translation [37], where centrin protein was no longer exclusively translated and assembled into basal bodies within spermatogenous cells, but instead, was synthesized and observed to aggregate into blepharoplast-like particles both in sterile jacket cells and spermatogenous cells within the spore wall [37].

Since Mv-Mago may also play a role in the asymmetric distribution of nuclear speckle components, including masked centrin mRNA, the effect of Mv-Mago silencing on the distribution of both masked mRNA and associated proteins was assessed. RNAi has previously been demonstrated to abolish detectable translation of Mv-Mago [37]. Gametophytes treated with dsRNA targeting Mv-Mago transcripts for RNAi were labeled with anti-U2B"antibody as a way to determine whether Mv-Mago and the EJC play a role in the asymmetric distribution of protein associated with masked mRNA in the nucleus. RNAi silencing of Mv-Mago resulted in a range of division anomalies described previously [37] and caused the symmetric distribution of U2B" protein to spermatogenous and jacket cells, as well as the disorganization of U2B" in spermatogenous cells (Figure 5s-5u: compare with Figure 5j-5l). We performed FISH on masked centrin transcripts after Mv-Mago silencing (Figure 5m-5o: compare with Figure 5d-5i). Like U2B" protein, masked centrin mRNA localized symmetrically to both spermatogenous and jacket cells (Figure 5m-5o). The effect of Mv-Mago silencing on the asymmetric distribution of masked SDPS transcripts was also assessed (Figure 5p-5r). Like with masked centrin transcripts (Figure 5m-5o) and U2B" protein (Figure 5s-5u), the loss of Mv-Mago resulted in the symmetric distribution of masked SPDS transcripts (Figure 5p-5r). These differences in transcript and protein distribution were also apparent in gametophytes that exhibited only minor anomalies in their cell division patterns after Mv-Mago silencing (Additional file 11; see [37]). Together, these results indicate that components associated with nuclear speckles become asymmetrically redistributed to the cytoplasm of spermatogenous cells by a mechanism dependent on Mv-Mago and likely to involve the EJC (compare localization of foci in spermatogenous cells (arrowheads) to localization of foci in jacket cells (arrows) in Figure 5d-5u). In addition, the subcellular localization of U2B" protein to the ends of elongating chromosomes appears to be mediated by Mv-Mago.

## Discussion

### Transcriptional silencing and dormancy triggers the coalescence of nuclear speckles and the storage of masked mRNA in the drying microspore of *M. vestita*

The male gametophyte of *M. vestita *relies on the regulated translation of stored mRNA for the rapid production of motile spermatozoids. The packaging of these transcripts for long-term storage in the spore is an essential mechanism required for rapid development leading to male gamete formation. In this paper, we have shown that nuclear speckles enlarge and aggregate as transcription is silenced in the desiccating microspore. In cells with blocked transcription, nuclear speckles often enlarge and serve as sites of storage for pre-mRNA processing machinery and under these conditions, a subset of poly(A)+ RNA is retained within the nucleus associated with nuclear speckles [18].

Transcription in the microspore remains silent even upon hydration and recovery from dormancy. We have shown that during spermatogenesis, nuclear speckles are maintained as a single coalescence in the nucleoplasm of the newly hydrated microspore. Morphologically, the nuclear speckle aggregate superficially resembles a nucleolus. However, the lack of colocalization with traditional nucleolar markers (Fibrillarin and rRNA) in the subnuclear aggregate reduces the likelihood of this possibility. Moreover, our studies of the desiccating microspore demonstrate a marked loss of nucleolar markers during microspore entry into quiescence. Like many dormant systems that stockpile polysomes [38-40], our findings show that a traditional nucleolus is absent from the desiccated microspore and suggest that little, if any, new ribosome biogenesis occurs during spermatogenesis in *M. vestita*.

U2B", which is present in the subnuclear aggregation, is commonly used as a marker of Cajal bodies (CBs) in both animals and plants. Is the aggregate a CB? Coilin is widely considered to be a diagnostic marker of the CB in animals [41,42], where it functions to concentrate and facilitate snRNP formation [43]. Unfortunately, coilin proteins in animals and plants are highly divergent, and they are sufficiently distinct so that anti-coilin antibodies used to identify CBs in animal cells show no specific affinity for plant coilins [44]. Despite the presence of U2B" within the subnuclear aggregate of *M. vestita*, no CBs examined have been shown to be associated with poly(A)+ RNA [for review, [45]. In addition to poly(A)+ RNA, CBs are known to lack SC35 [46]. Using the lack of poly(A)+ RNA and SC35 as criteria for CB identity, it does not appear that the subnuclear aggregation present in the microspore should be designated as a CB since it contains both polyadenylated RNA and the essential splicing factor SC35.

Nuclear speckles contain U2B" and SC35, and a subset of poly(A)+ RNA. In addition to similarities in composition, the behavior of the aggregated nuclear material in the microspore of *M. vestita *resembles traditional nuclear speckles more closely than other kinds of nuclear inclusions. Typically, nuclear speckles consist of small, interchromatin aggregations of pre-mRNA splicing proteins, but have been shown to enlarge upon transcriptional inhibition [18]. This enlargement apparently results from an accumulation of pre-mRNA splicing machinery in the speckles and these stores are utilized once transcription is reinitiated. In addition to the accumulation of pre-mRNA splicing machinery, previous work has shown that transcriptional inhibition causes a subset of poly(A)+ RNA to be sequestered within nuclear speckles [18]. Our observations of desiccating microspores suggest that a similar pattern occurs; TBO and PY staining as well as SC35 antibody labeling reveal that small aggregations coalesce into larger accumulations, finally resulting in a single large aggregation concurrent with the onset of transcriptional quiescence. The similarities between aggregated SC35, U2B" and poly(A)+ RNA in the microspore of *M. vestita *and traditional speckles are inescapable. Beyond the storage of pre-mRNA processing machinery, nuclear speckles could serve as sites for the storage of mRNA during periods of inhibited transcription and/or splicing. Intriguingly, a subset of speckle associated poly(A)+ RNA is masked mRNA (SPDS and centrin) that is known to be essential for gametophyte development in *M. vestita*. It is important to point out that with the required use of Proteinase K in our *in situ *hybridization protocols, FISH and immunofluorescence labeling had to be performed sequentially as described in the Methods section. Immunofluorescence labeling had to be performed first, followed by imaging and removal of coverslips, prior to treating the cells for FISH. We attempted a number of methods that would allow simultaneous immuno- and in situ labeling on the same sections of gametophytes, but we had to resort to sequential labeling in order to obtain reproducible patterns of antibody and RNA distributions in the cells. The sequential method used has the unfortunate drawback that in between imaging sections can become distorted because of the harsh incubation conditions for FISH or ISH. We feel that this is why in all such experiments (Figure 2b, d, f, h) subnuclear poly(A)+ RNA appears to be more diffuse than immunofluorescent labeling (Figure 2a, e) or nuclear poly(A)+ labeling alone (Figure 4b, d).

### Asymmetric distribution of nuclear speckle components to the cytoplasm of spermatogenous cells

Nuclear speckles remain aggregated within the nucleus of the microspore until the first division. During the first division, both protein and RNA associated with nuclear speckles enter the cytosol adjacent to the nucleus of the antheridial initial. As additional division cycles progress, this material is asymmetrically distributed to spermatogenous cells, but not to jacket cells. During spermatogenesis, foci of masked transcripts were visible in the cytoplasm directly adjacent to the nuclei of spermatogenous cells, but were not seen in jacket cells. Sectioned material was used for the analysis of this asymmetric masked transcript distribution, because FISH and immunofluorescence assays cannot be performed together on intact, but fixed spores, even if they are imaged by confocal microscopy. Thus, while the foci of masked centrin and SPDS RNAs should not be expected to be visible in all of the spermatogenous cells in a single section of a single gametophyte, observations of many sections of many gametophytes provide strong indications that all of the spermatogenous cells contain these foci of masked transcripts.

While the ISH and FISH assays carried out in this study were not directed against RNA contained exclusively within jacket cells, previous studies (15) have demonstrated that the techniques used here are capable of labeling transcripts contained exclusively within jacket cells. In addition Figure 5f, i, o and 5r shows the labeling of jackets cells, confirming their accessibility to our probes and hybridization techniques.

There are two critical co-variants in gametophyte development: a) the patterns of divisions leading to the formation of sterile and spermatogenous cells are precise inside the spore wall, and b) there is no cell movement during development. These factors highlight an essential process in cell fate determination in the gametophyte: the fragmentation of aggregated nuclear speckle-associated masked transcripts must be orderly to ensure that appropriate complements of mRNAs are distributed among all of the spermatids. The movements of masked transcripts must be under strict control during the successive division cycles, so that appropriate parcels of transcripts are ultimately allocated among the 32 spermatids in the gametophyte. Thus, masked mRNA, originally stored in a single coalescence of nuclear speckles within the nucleus of the desiccated microspore, is dispersed as a set of masked RNA-containing particles that are uniformly distributed among spermatogenous cells, but not to sterile jacket cells, which arise from the same progenitors.

The EJC exerts profound effects on the symmetry of divisions in the developing gametophyte of *M. vestita *[37]. By silencing EJC components, we induced perturbations of division plane locations that effectively disrupted spermatid-specific events. Here, we show that dsRNA-mediated silencing of Mv-Mago also disrupted the asymmetric distribution of centrin masked mRNA and U2B" protein (Figure 5, Additional file 11). Mv-Mago clearly mediates the cell type-specific distribution of stored nuclear transcripts.

In other plants, the EJC and in particular Mago nashi have been shown to be essential for development in nearly all tissues and organs. The silencing of atMago in *Arabidopsis *[33] results in aberrant microspore tetrad arrangement. eIF4A-III, another core component of the EJC relocalizes during stress [31,32] in nucleoli and nuclear speckles. When present in nuclear speckles, eIF4A-III displays lowered mobility suggesting that it is specifically retained within these subdomains [31]. The presumed consequence of this relocalization is that mRNA associated with eIF4A-III would not exit the nucleus and thus its translation would be inhibited [31,32]. We believe a similar mechanism could be at work in the microspores of *M. vestita *where mRNA is stored during desiccation in association with nuclear speckles. We propose that the association of this RNA with these speckle components ensures nuclear retention that in turn, forestalls its translation.

### A mechanism for cell type-specific translation of centrin

Mv-Mago silencing results in the symmetric distribution of masked centrin mRNA as well as the symmetric translation of centrin in both spermatogenous and jacket cells at 4 hours of development. Masked centrin mRNA initially stored in the nucleus of the microspore is clearly required for centrin protein production in the spermatogenous cells of the gametophyte. It follows that additional levels of translational regulation must be in place to inhibit the translation of cytoplasmic centrin mRNA, which is ubiquitously present in all cells at all times during spermatogenesis [12]. Although qc-centrin transcripts are distributed throughout the cytosol of all cells in the gametophyte from the onset of development, translation of centrin protein occurs only after 4 hours of development, and only within spermatogenous cells. In addition, previous research from our laboratory [15] has shown that masked subnuclear RNA that is initially undetectable using long *in situ *hybridization probes later becomes detectable with these long probes in the cytoplasm of spermatogenous cells at time points when the corresponding proteins become abundant [15]. When combined with information from *in vitro *translation assays [8] that show RNA isolated from these spores cannot be translated prior to the first division of the gametophyte (when subnuclear transcripts are apparently released from the nucleus), these data suggest not only that masked subnuclear mRNA plays an important role in spermatogenesis, but also that cytoplasmic stores of RNA remain translationally quiescent during gametophyte development. The differential regulation of translation for masked RNA and quiescent cytoplasmic transcripts is not fully apparent at this time, but an association with nuclear speckles appears to be essential for the translation of centrin mRNA, and probably other proteins as well. Further elucidation of this mechanism(s) will be essential for our understanding fate determination in the highly ordered and transcriptionally quiescent gametophyte.

### Future perspectives

The discovery of a coalesced nuclear speckle aggregate, whose function includes the storage and masking of developmentally important transcripts, reveals an important level of post-transcriptional regulation affecting rapid development of spermatozoids in *M. vestita*. We believe this coalescence contributes substantively to the long-term storage of stable, masked RNAs that may be present as fully or partially processed transcripts, which are essential for the formation of spermatozoids within hours after the dry microspore is hydrated. Furthermore, it is apparent that the mode of storage of these transcripts may play a role in their translational regulation and their asymmetric distributions during spermatogenesis.

Paraspeckles, subnuclear aggregates closely associated with nuclear speckles [47] retain CTN-RNA, which is a non-coding mCAT2 transcript [48]. Under stress, CTN-RNA is cleaved, thereby releasing protein-encoding mCAT2 mRNA, which is quickly localized to the cytosol for translation [48]. Our finding that nuclear speckles participate in the storage of masked mRNA builds on the paradigm that the nuclear retention of transcripts plays an important role in gene expression.

Many subnuclear bodies (including nuclear speckles) have a cytoplasmic phase in their cycles [49,16,52] and there is mounting evidence that the displacement of some bodies to the cytoplasm plays a key role in cellular function. For example, the nucleolinus of *Spisula *oocytes enters the cytoplasm following activation, and centrosomes form within the diffusing nucleolinus. In parthenogenetically activated oocytes, laser ablation of the nucleolinus results in a failed meiotic division and microtubule disorganization, demonstrating a clear functional role for the nucleolinus in spindle formation and cell division [52]. We suspect that in addition to the storage of masked mRNA, nuclear speckles could serve to regulate the asymmetric distribution and translation of these messages in the cytoplasm of spermatogenous cells.

Ongoing and future characterizations of the subnuclear masked mRNA and associated processing machinery should provide greater insights into regulatory mechanisms that underlie this rapid developmental process. We believe that traditional 'long' ISH or FISH probes could not detect masked mRNA because masking agents (most likely proteins) obscure sites of hybridization. We believe because of their small size our 25mer probes can be fit in between their masking agents and thus robustly label masked mRNA. We would like to identify these masking agents and further assess their role(s) in development and RNA regulation. An obvious area of interest centers on the fragmentation process and the distribution of masked transcripts among the spermatids. Since both centrin and SPDS masked transcripts become localized exclusively in foci within spermatogenous cells, these transcripts can reveal patterns of storage, movement and unmasking for particular mRNAs during development. Our FISH assays show that transcript pools may be stored discretely, so how and when are these particles fragmented and passed on to spermatogenous cells? If masked transcripts are stored in multiple foci, how does each spermatid receive a full complement of masked transcripts? How is the timing of translation controlled for specific transcripts during development? The mechanisms that control how the subnuclear RNA and pre-mRNA processing machinery becomes asymmetrically distributed between spermatogenous and sterile cells remains unclear. Positioning of the subnuclear material in the cytoplasm during the prothallial division clearly underlies this process, and identifying the components and factors that affect movements of these molecules will be important in understanding cell fate determination mechanisms in the highly ordered gametophyte.

## Conclusions

We show here that translationally masked RNA is stored within aggregated nuclear speckles during desiccation and dormancy in the microspore of *M. vestita*. In addition, we show that both protein and nucleic acid components of this speckle aggregate are asymmetrically localized to spermatogenous cells during development. This localization requires the EJC core component Mago nashi. Asymmetric localization of masked mRNA mirrors both the temporal and spatial patterns of their corresponding proteins. We believe this suggests a role for nuclear speckles in the post-transcriptional regulation of mRNA.

## Methods

### Microspore culture and fixation

*Marsilea vestita *microspores were obtained, cultured, fixed, and sectioned as previously described [53,9].

### Microscopy

All widefield fluorescence microscopy was performed with a Zeiss Axioscope equipped with standard Fluorescein, TexasRed and UV filter sets. Confocal microscopy was performed on a Zeiss LSM700 using Zen 2009 software. Subsequently, .lsm stacks were exported into ImageJ 1.44k and rendered as 3D models using the ImageJ 3D Viewer plugin.

### DAPI Pyronin Y double staining

DAPI and pyronin Y are available from a number of commercial sources. DAPI was diluted in PBS and used at a final concentration of 2.5 μg/ml. Pyronin Y was used at 5% in deionized water. Pyronin Y (5%) was added 1:1 with 0.2M pH 4 acetate buffer on the day of use to make PY staining solution. Slides with sectioned material were incubated in acetone for 15 minutes on a rocker. Slides were next incubated in PBS for five minutes; this was repeated twice. Samples were placed in a humid chamber and incubated with DAPI for 10 minutes followed by incubation in PBS-T for five minutes and then PBS for five minutes on a rocker. Slides were incubated in a humid chamber for 1.5 minutes with PY staining solution, rinsed with deionized water and dehydrated in an ethanol series (1 minute incubations at 25%, 50%, 75%, 90%, 100%, and 100%). Slides were dipped briefly in xylene.

### ISH and FISH probe production

Probes for 5S and 25S rRNA were constructed through digoxigenin-11-dUTP incorporation as previously described [11]. ISH probes against cytoplasmic (qc) centrin and SPDS transcripts were made as follows. cDNA constructs encoding both centrin and SPDS were obtained from our cDNA library [8,13]. Single stranded antisense probes were transcribed using T7 RNA polymerase. Probes were biotinylated using the PHOTOPROBE biotin kit for nucleic acid labeling (Vector Laboratories http://www.vectorlabs.com/). Biotinylated poly(T) probe was purchased from GibcoBRL. 25mer biotinylated DNA probes for the detection of masked centrin and SPDS transcripts were obtained through the custom DNA oligo service at Integrated DNA Technologies http://www.idtdna.com.

### ISH and FISH detection

The ISH protocol used here has been previously described [11]. Detection of biotinylated FISH probes was achieved through the use of a goat-anti-biotin antibody, followed by a rabbit-anti-goat antibody conjugated with AlexaFluor 594. Alternatively, biotinylated probes requiring signal amplification were detected using either TexasRed or Fluorescein labeled Avidin (Vector Laboratories A-2016 and A20-11) followed by biotinylated anti-Avidin D (Vector Laboratories BA-0300) labeling and a second round of TexasRed or Fluorescein labeled Avidin labeling following the manufacturers instructions.

### Fluorescence *in situ *hybridization of masked transcripts

FISH on masked transcripts was performed as detailed previously [11] with the following modifications. Hybridization was carried out for 16 hours at 30°C. Detection was carried out using avidin/biotin signal amplification reagents (Vector Laboratories) as described above. In double labeling experiments, hybridization and detection of one probe was carried out, followed by avidin/biotin blocking (Vector Laboratories SP-2001), and subsequent hybridization and detection of the second probe.

### Cytology and Immunocytochemistry

Toluidine Blue O staining [54] was performed on sectioned material and observed via bright-field microscopy. DAPI staining was performed as described previously [37]. Immunolabeling of sectioned material was carried out was described by Baskin and Wilson [55] with modifications described by van der Weele *et al*. [37]. Antibodies used in this study were as follows: 4G3 mouse monoclonal antibody against U2B" and 72B9 mouse monoclonal antibody against Fibrillarin (generously provided by Dr. Joseph Gall Carnegie Institution of Washington, Baltimore, MD) and were used at dilutions of 1:5, and 1:10 respectively. Mouse monoclonal antibody against SC35 was obtained commercially from Abcam (ab11826 http://www.abcam.com) and used at a dilution of 1:200. Goat-anti-biotin, AlexaFluor 594 conjugated goat-anti-mouse and AlexaFluor 594 conjugated rabbit-anti-goat antibodies were obtained from Molecular Probes (Molecular Probes, Invitrogen Detection Technologies http://www.invitrogen.com) and were used at 1:200, 1:1000, and 1:1000 dilutions respectively. Histochemical detection of antibodies was carried out using an alkaline phosphatase conjugated anti-mouse antibody, followed by visualization with nitro-blue tetrazolium and 5-bromo-4-chloro-3-indolyl phosphate.

### Immunocytochemistry/FISH double labeling

In double labeling experiments, antibody labeling was performed as described above, coverglasses were mounted but not sealed. Following imaging, coverglasses were removed by incubating slides in PBS-T, 3 times for 5 minutes on a rocker. Following coverglass removal, our standard FISH protocol was followed as detailed above.

### RNAi

RNA interference was performed as described by Klink and Wolniak [9]. Briefly, a cDNA clone encoding Mv-Mago was obtained from our cDNA library [8,13]. Single stranded RNA was transcribed from this clone using T7 and T3 RNA polymerases. Single stranded RNA was annealed in vitro. Microspores were placed 1 mL of spring water with 200 μg/mL double stranded RNA and allowed to develop for desired periods of time, fixed, and sectioned as described above and previously.

### Polyamine treatment

10 mM Spermidine treatments were performed as previously described [15].

## Authors' contributions

TCB performed experiments, participated in the design of experiments and interpretation of the data, and writing of the manuscript. SMW participated in the design of experiments, the interpretation of data, and the writing of the manuscript. TCB and SMW both read and approved the final manuscript.

## Supplementary Material

Additional file 1 **Table S1. Numbers of independent trials and representative photographs for experiments presented**. Column 1 lists the experiment(s). Column 2 lists the figure(s) with representative images from specific experiment(s). Column 3 lists the total number of photographs taken for each experiment(s). Column 4 lists the total number of independent trials for each experiment(s). Click here for file

Additional file 2 **Figure S1. Differential fluorescent labeling of DNA and RNA via dual DAPI/PY staining**. Microspores fixed at 30 minutes of development. PY (green) (**a**, **c**, **e**, **g**, **i**, **k**) and DAPI (blue) (**b**, **d**, **f**, **h**, **j**, **l**) detected via 488 nm and UV illumination respectively. **a**-**b**, Microspore stained with PY (green). **c**-**d**, Microspore stained with DAPI (blue). **e-f**, Microspore double-stained with DAPI (blue) and PY(green). **g**-**h**, Microspore pretreated with RNase and double-stained with both DAPI (blue) and PY (green). **i**-**j**, DNase pretreated samples were double-stained with DAPI (blue) and PY (green). **k**-**l**, DNase and RNase treated samples were double-stained with DAPI (blue) and PY (green). Bar = 25 μm. Click here for file

Additional file 3 **Movie S1. Pyronin Y labeling reveals discreet aggregations of subnuclear RNA after 2 weeks of dehydration**. Microspores collected and fixed after 2 weeks without watering were sectioned and double stained with DAPI and PY. Subnuclear PY (green) signal was detected in successive confocal slices and these were rendered as a 3D model. Movie is in .mov format playable with QuickTime. Click here for file

Additional file 4 **Movie S2. Pyronin Y labeling reveals a partial coalescence of RNA aggregates after 4 weeks of dehydration**. Microspores collected and fixed after 4 weeks without watering were sectioned and double stained with DAPI and PY. Subnuclear PY (green) signal was detected in successive confocal slices and these were rendered as a 3D model. Movie is in .mov format playable with QuickTime. Click here for file

Additional file 5 **Movie S3. Pyronin Y labeling reveals total coalescence of RNA aggregates after 6 weeks of dehydration**. Microspores collected and fixed after 6 weeks without watering were sectioned and double stained with DAPI and PY. Subnuclear PY (green) signal was detected in successive confocal slices and these were rendered as a 3D model. Movie is in .mov format playable with QuickTime. Click here for file

Additional file 6 **Figure S2. RNA is not detectable within the nuclei of microspores after the first division**. **a**-**c**, Microspore fixed and sectioned after 1.5 hours of development. **a**, DAPI (blue). **b**, Pyronin Y (green). **c**, merge of **a **and **b**. Bar = 25 μm. Click here for file

Additional file 7 **Movie S4. Spermidine additions cause the partial dissociation of aggregated nuclear RNA**. Microspores were incubated with 10 mM SPD for 4 hours, fixed, sectioned and double stained with DAPI and PY. Subnuclear PY (green) and DAPI (blue) signals were detected in successive confocal slices and these were rendered as a 3D model. Movie is in .mov format playable with QuickTime. Click here for file

Additional file 8 **Movie S5. Spermidine additions cause the total dissociation of aggregated nuclear RNA**. Microspores were incubated with 10 mM SPD for 4 hours, fixed, sectioned and double stained with DAPI and PY. Subnuclear PY (green) and DAPI (blue) signals were detected in successive confocal slices and these were rendered as a 3D model. Movie is in .mov format playable with QuickTime. Click here for file

Additional file 9 **Movie S6. 'Short' FISH probes detect foci of subnuclear masked transcripts that are distinct from chromatin**. FISH against masked SPDS (red) was conducted on 20 μm sections taken from microspores fixed after 30 minutes after hydration. Probes were detected using avidin bound TexasRex. Sections were counterstained with DAPI (blue). Movie is in .mov format playable with QuickTime. Click here for file

Additional file 10 **Figure S3. Traditional FISH probes fail to detect masked SPDS transcripts within the nuclei of maturing microspores**. **a**-**c**, Traditional biotinylated probes directed against SPDS transcript (red). Pre-prothallial (**a**), mid-prothallial (**b**), and late-prothallial (**C**) division microspore. The prothallial nucleus denoted by "p" in **b **and **c**. Bar = 5 μm. Click here for file

Additional file 11 **Figure S4. Defects in asymmetric division are not requisite for symmetric distribution of subnuclear material in Mv-Mago knockdowns**. **a**-**c**, microspore subjected to Mv-Mago RNAi and fixed after 5 hours of development. (**a**) DAPI (blue), (**b**) phase contrast, (**c**) 4G3 labeling of U2B" (red). **d-f**, representative microspore subjected to Mv-Mago RNAi and fixed after 4 hours of development. (**d**) DAPI (blue), (**e**) phase contrast, (**f**) masked centrin transcripts (red) detected with 25mer biotinylated FISH probes. Spermatogenous cells denoted by "sp" and jacket cells denoted by "jk." Bar = 25 μm. Click here for file
