# Supplementary material for: Masked mRNA is stored with aggregated nuclear speckles and its asymmetric redistribution requires a homolog of mago nashi
Source: BMC Cell Biol. 2011 Oct 13;12:45. doi: 10.1186/1471-2121-12-45 (PMC3205038; doi:10.1186/1471-2121-12-45)
Supplement: Additional file 1 — Table S1. Numbers of independent trials and representative photographs for experiments presented. Column 1 lists the experiment(s). Column 2 lists the figure(s) with representative images from specific experiment(s). Column 3 lists the total number of photographs taken for each experiment(s). Column 4 lists the total number of independent trials for each experiment(s). [file 1471-2121-12-45-S1.DOCX]

| **Experiment** | **Figure #** | **Total image count** | **Independent trials** |
| --- | --- | --- | --- |
| Labeling of desiccating microspores | Figure 1 | 172 | 8 |
| SC35 labeling | Figure 2 | 50 | 2 |
| U2B" labeling | Figure 2, 5 | 78 | 2 |
| Fibrillarin labeling | Figure 2 | 41 | 2 |
| rRNA ISH | Figure 2 | 62 | 2 |
| PY/DAPI Staining | Figure 3, Supplemental Figure 1, 2 | 428 | 16 |
| Poly(A) ISH | Figure 3 | 45 | 2 |
| Masked mRNA FISH | Figure 3, 4, 5, Supplemental Figure 3 | 403 | 10 |
| Mago knockdown FISH and IF | Figure 6, Supplemental Figure 4 | 138 | 6 |
