# Supplementary figures and images for: Masked mRNA is stored with aggregated nuclear speckles and its asymmetric redistribution requires a homolog of mago nashi
Source: BMC Cell Biol. 2011 Oct 13;12:45. doi: 10.1186/1471-2121-12-45 (PMC3205038; doi:10.1186/1471-2121-12-45)

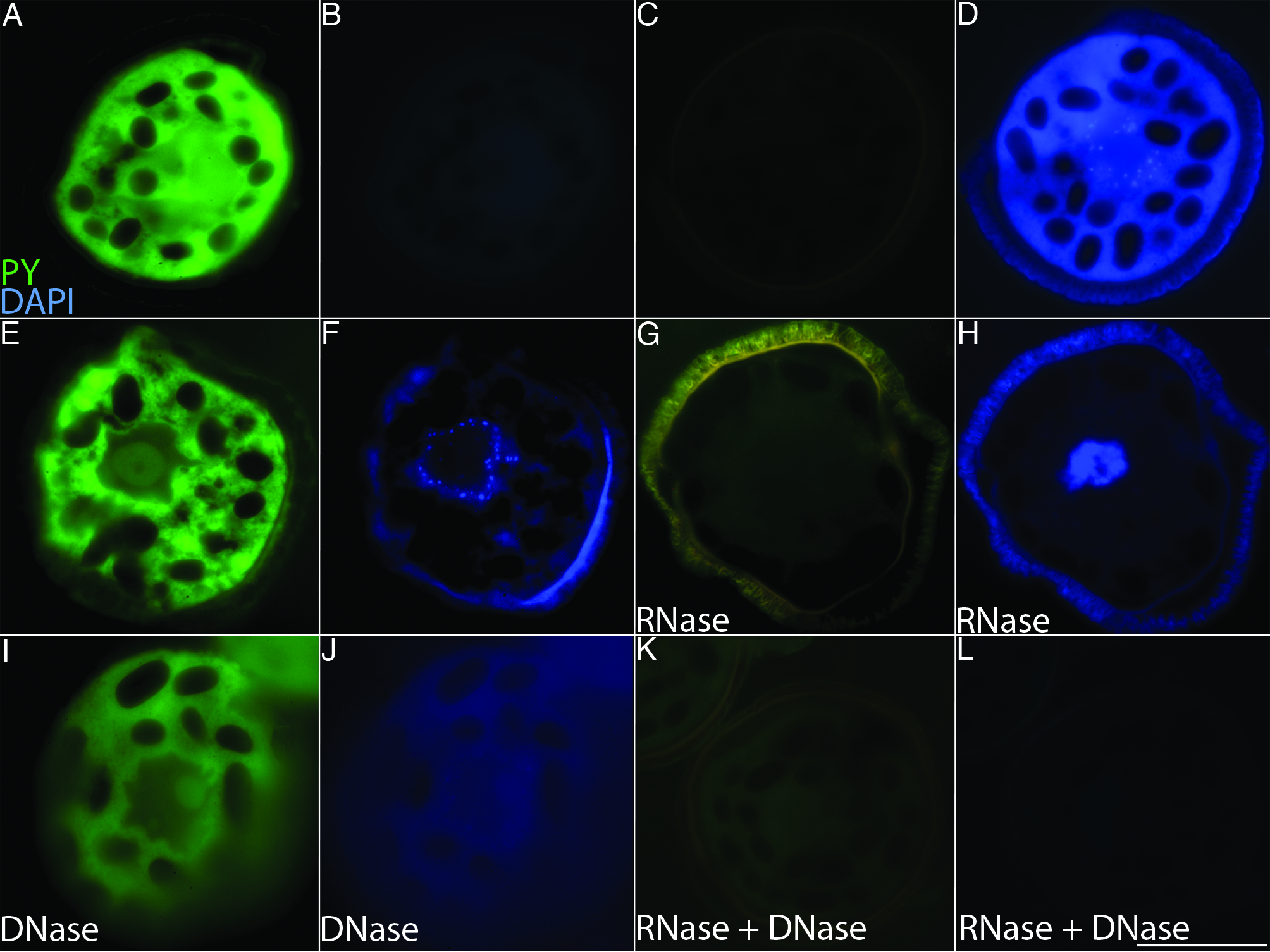

Supplement: Additional file 2 — Figure S1. Differential fluorescent labeling of DNA and RNA via dual DAPI/PY staining. Microspores fixed at 30 minutes of development. PY (green) (a, c, e, g, i, k) and DAPI (blue) (b, d, f, h, j, l) detected via 488 nm and UV illumination respectively. a-b, Microspore stained with PY (green). c-d, Microspore stained with DAPI (blue). e-f, Microspore double-stained with DAPI (blue) and PY(green). g-h, Microspore pretreated with RNase and double-stained with both DAPI (blue) and PY (green). i-j, DNase pretreated samples were double-stained with DAPI (blue) and PY (green). k-l, DNase and RNase treated samples were double-stained with DAPI (blue) and PY (green). Bar = 25 μm. [file 1471-2121-12-45-S2.TIFF]

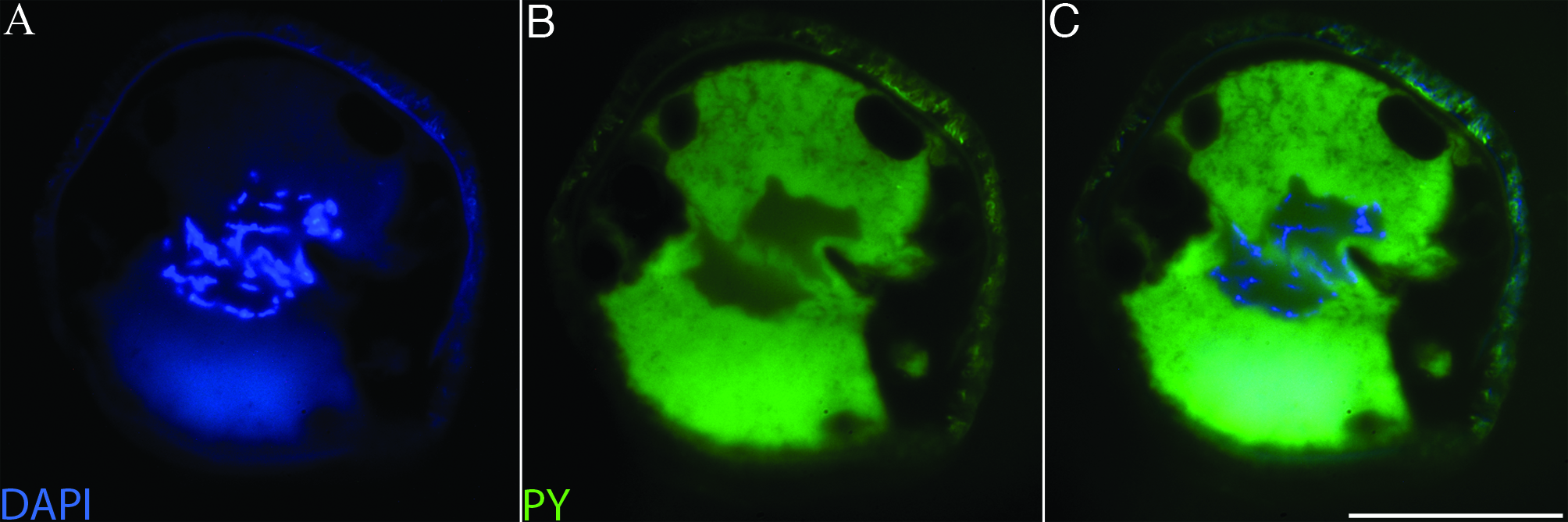

Supplement: Additional file 6 — Figure S2. RNA is not detectable within the nuclei of microspores after the first division. a-c, Microspore fixed and sectioned after 1.5 hours of development. a, DAPI (blue). b, Pyronin Y (green). c, merge of a and b. Bar = 25 μm. [file 1471-2121-12-45-S6.TIFF]

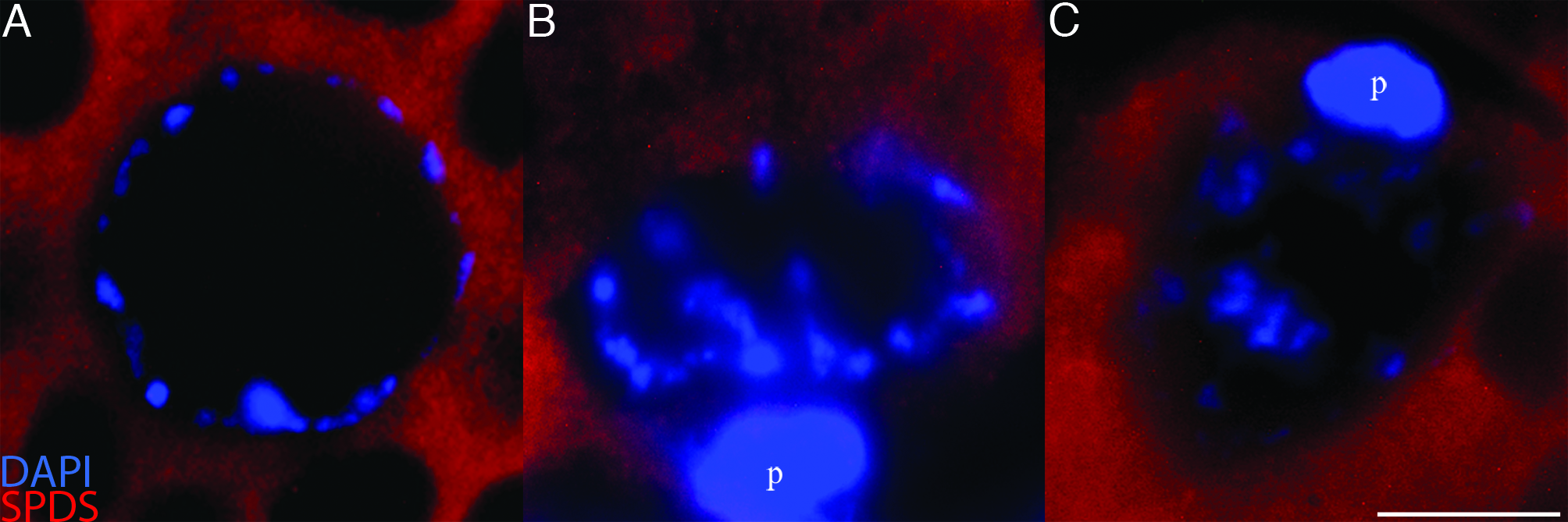

Supplement: Additional file 10 — Figure S3. Traditional FISH probes fail to detect masked SPDS transcripts within the nuclei of maturing microspores. a-c, Traditional biotinylated probes directed against SPDS transcript (red). Pre-prothallial (a), mid-prothallial (b), and late-prothallial (C) division microspore. The prothallial nucleus denoted by "p" in b and c. Bar = 5 μm. [file 1471-2121-12-45-S10.TIFF]

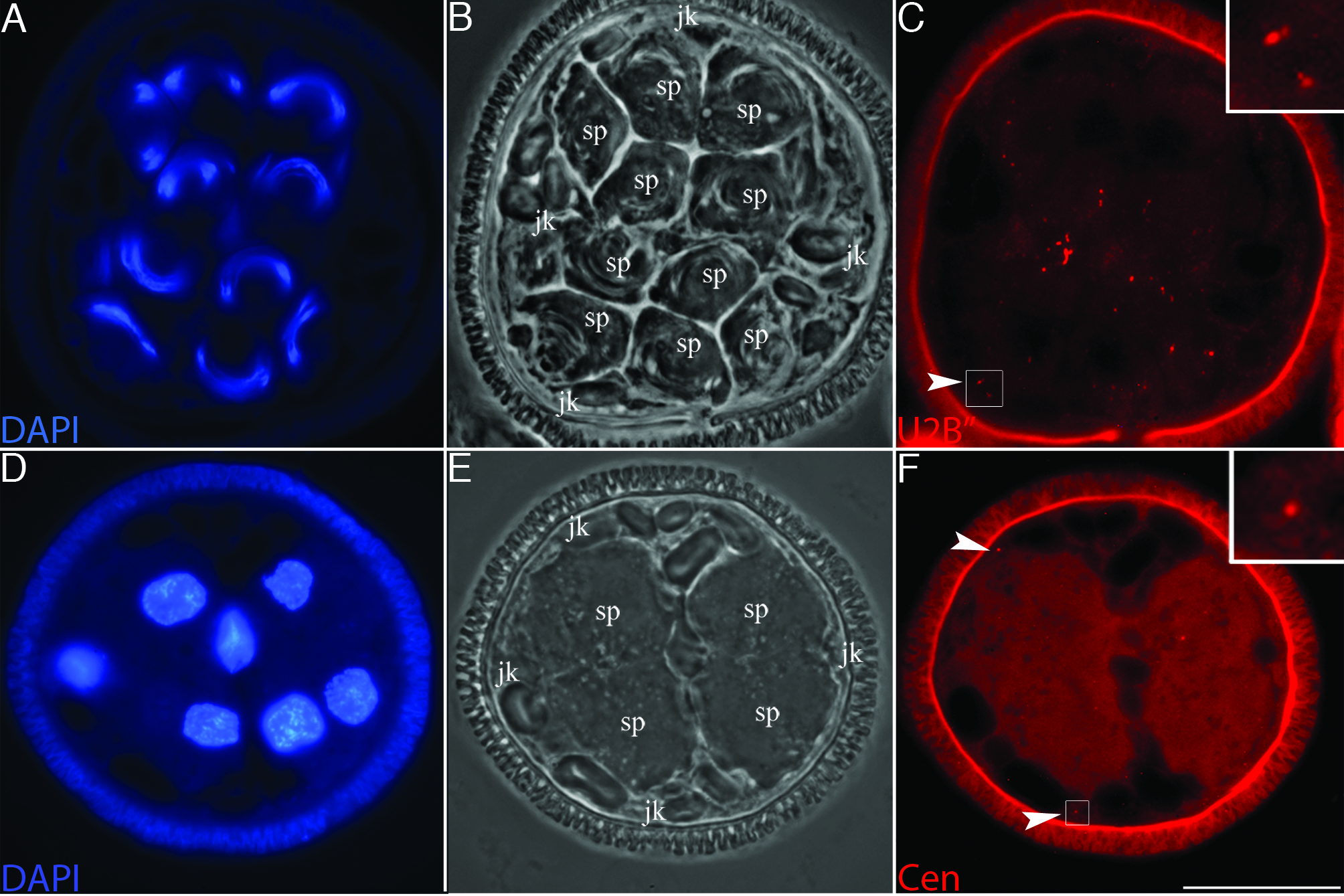

Supplement: Additional file 11 — Figure S4. Defects in asymmetric division are not requisite for symmetric distribution of subnuclear material in Mv-Mago knockdowns. a-c, microspore subjected to Mv-Mago RNAi and fixed after 5 hours of development. (a) DAPI (blue), (b) phase contrast, (c) 4G3 labeling of U2B" (red). d-f, representative microspore subjected to Mv-Mago RNAi and fixed after 4 hours of development. (d) DAPI (blue), (e) phase contrast, (f) masked centrin transcripts (red) detected with 25mer biotinylated FISH probes. Spermatogenous cells denoted by "sp" and jacket cells denoted by "jk." Bar = 25 μm. [file 1471-2121-12-45-S11.TIFF]
